# The Long-Term Progression of Aneurysmal Disease in Common Iliac Arteries After Standard EVAR and Its Clinical Implications

**DOI:** 10.1155/ijvm/4229582

**Published:** 2024-11-29

**Authors:** Apostolos G. Pitoulias, Dimitrios Chatzelas, Matthaios G. Pitoulias, Loukia A. Politi, Dimitrios C. Christopoulos, Ioannis Lazaridis, Nikolaos Saratzis, Georgios A. Pitoulias

**Affiliations:** ^1^Second Department of Surgery, Division of Vascular Surgery, “G. Gennimatas” General Hospital of Thessaloniki, School of Health Sciences, Faculty of Medicine, Aristotle University of Thessaloniki, Thessaloniki, Greece; ^2^First Department of Surgery, Division of Vascular Surgery, “G. Papageorgiou” General Hospital of Thessaloniki, School of Health Sciences, Faculty of Medicine, Aristotle University of Thessaloniki, Thessaloniki, Greece

**Keywords:** aortic aneurysm repair, distal iliac migration, distal sealing zone, limb retraction, long-term surveillance, Type Ib endoleak

## Abstract

**Introduction:** The progression of aneurysmal disease in the common iliac arteries (CIAs) after EVAR remains an insufficiently investigated field. The purpose of this study is to investigate the long-term outcomes of standard elective EVAR with a variety of last-generation bifurcated aortic endografts in relation with the progression of aneurysmal disease in the CIAs.

**Methods:** This is a prospective cohort study of 168 patients, who were treated with six different endografts between 2013 and 2018 and completed the 5-year computed tomography aortoangiography (CTA) follow-up. Postoperative CTA analysis included CIA measurements at four diameters' points and two length levels in three postoperative time spots: first, 24th, and 60th months. All EVAR-related adverse events were recorded, including migrations, endoleaks, limb occlusions, reinterventions, ruptures, and mortality.

**Results:** At both time intervals, a significant and nearly linear dilatation and elongation of CIAs was evident. The mean percent increase, among all diameter points measured, was 11.7% at 24 months and 22.8% at 60 months (*p* < 0.001) with a nearly constant mean increase rate by 0.07 mm per month. The corresponding monthly elongation rate of total CIA length was 0.26 mm at 24 months and 0.34 mm at 5 years (*p* < 0.001). The respective monthly lengthening of CIAs' uncovered (from stent graft) segment was 0.10 and 0.15 mm, and the overall increase rate at 60 months was up to 53.9% (*p* < 0.001). A total of 20 EVAR-related events were recorded, and multivariate analysis revealed that CIA dilatation served as a significant and independent predictor of long-term EVAR failures, increasing the likelihood of adverse events by 2.8-fold.

**Conclusions:** Analysis of long-term geometric CIA remodeling after a standard EVAR revealed a significant progression of aneurysmal disease in CIAs, which was associated with worsening EVAR outcomes and emphasizes the importance of a rigorous and extensive follow-up protocol to maintain the long-term EVAR effectiveness.

## 1. Introduction

Abdominal aortic aneurysm (AAA) repair by traditional open (OR) or endovascular (EVAR) techniques remains the biggest “chapter” in vascular surgery, and related research is always at the center of interest from both the scientific community and industry.

Early concerns about the effectiveness of EVAR were first answered by the DREAM trial which showed superiority of EVAR over the OR in 30-day operative mortality and graft-related complications and reinterventions [1, 2]. However, the degenerative nature of the underlying pathophysiology which continues to affect the aortoiliac vessels' wall and the continued exposure to predisposing factors over time, even after the implantation of the stent graft, compromise the long-term outcomes of EVAR [3]. The outcomes of the UK EVAR trial showed increased rates of late-onset EVAR-related complications and reinterventions which, even after an early perfectly effective EVAR, appear to be attributable to the long-term hemodynamic strains and to the slow but surely present post-EVAR continuous remodeling of the aortoiliac vessels over time [4–6].

The most common problems and greater pitfalls that may be encountered in a long-term failed EVAR are primarily related to loss of sealing at the proximal aortic and distal iliac landing zones, resulting in late endoleaks and/or migration of the stent graft [7]. To date, several studies, including meta-analyses, have been published highlighting the post-EVAR failure due to dilatation of the proximal aortic neck and loss of sealing in this landing zone [8–10]. In addition, numerous studies have investigated the preoperative anatomical features of the proximal aortic neck and its dilatation over time, providing a better understanding of the long-term hemodynamic alterations at this anatomical level as well as information toward the possible prevention of this phenomenon by establishing stricter preoperative anatomical indications, which would ensure the long-term success of EVAR [11, 12].

In contrast with the proximal aortic neck, the phenomenon of long-term distal landing zone failure has been investigated only sporadically, by a few small case series studies with limited follow-up, so that the image of post-EVAR iliac artery remodeling as well as its correlation with EVAR failures, due to Type I endoleaks and stent graft migrations, remains unclear. The aim of this study is to document the post-EVAR, long-term, alterations of anatomical characteristics of the common iliac arteries and to investigate their potential association with graft failures.

## 2. Methods

The present study is a noninterventional prospective cohort study including data from patients treated between 2013 and 2018 at a single tertiary university vascular surgery center. The research protocol was approved by the institutional ethics committee and was in accordance with the principles set out in the Declaration of Helsinki. All patients gave their informed consent, which covered their participation and the storage and analysis of their medical information within the framework of this study, as well as for the potential publication of its results.

Our protocol included all consecutive patients with true fusiform, nonsymptomatic AAA, ≥ 55 mm in maximum sac diameter, or ≥ 50 mm in the rare occasions of rapid expansion rate (> 5 mm/year), and/or female gender, which were treated in our department technically successful at 30 days of elective standard EVAR, with a CE-marked contemporary bifurcated self-expanding abdominal aortic stent graft [13]. Clinical criterion for offering EVAR treatment was age above 65 years or coexistence of severe comorbidities for younger patients. Anatomo-morphological exclusion criteria for elective standard EVAR included patients with hostile proximal aortic neck, such as length < 12 mm, width or bulge > 30 mm, angulation > 60°, conical anatomy and circumferential thrombus or calcification > 50%, and/or hostile distal iliac neck anatomy with diameter ≥ 24 mm [12]. All standard EVAR procedures were performed by a single vascular surgeon, using the same protocol in preoperative planning, operative technique, and fluoroscopy equipment, exclusively with femoral cut-downs and with preference in locoregional anesthesia [14]. A total of 231 consecutive patients met the above standard elective EVAR criteria, and the stent graft selection was based on the surgeon's preference based on patient's individual anatomo-morphological features of the AAA and the availability of endograft devices in our hospital. The following six bifurcated self-expanding stent graft devices were used in patients included in the study: Endurant *ΙΙ* (Medtronic, Santa Rosa, California, United States), Anaconda (Terumo Aortic, Bolton Medical, Florida, United States), Treo (Terumo Aortic, Bolton Medical, Florida, United States), E-tegra (Artivion, Georgia, United States), AFX2 (Endologix, California, United States), and Ovation (Endologix, California, United States). From these stent grafts, the nitinol self-expanding scaffold has a fabric liner made by ePTFE in the AFX2 and Ovation devices and by Dacron (woven polyester) in the remaining four (Endurant *ΙΙ*, Anaconda, Treo, and E-tegra). All stent grafts have suprarenal fixation with uncovered bare metal stent crown except for Anaconda which offers infrarenal fixation only. All four woven polyester stent grafts offer additional proximal fixation with hooks.

All patients followed a common rigorous follow-up protocol for at least 5 years after EVAR, with clinical evaluation and color duplex ultrasound (cDUS) examination every 6 months as well with CTA imaging at the first postoperative month to verify the 30-day technical success and at 1, 2, and 5 years in order to detect any follow-up adverse outcome including endoleak, migration, stent graft fatigue, or occlusion as well to assess the aortic and iliac artery remodeling and to quantify the aortoiliac anatomo-morphological changes over time. In any case of evidenced or suspected by clinical or cDUS means of adverse outcome during follow-up, we performed interim CTAs.

Enrollment of patients for the analysis protocol of this study, from the pool of all 231 elective standard EVAR procedures, required the fulfillment of the following two simple criteria. The first was the 30-day technical success, which was defined as survival at 30 days, with patency of the stent graft and CTA evidence of absence of Types I, III, and IV endoleaks, migration, or any other EVAR-related complication. The second criterion was the completion of at least 60 months of follow-up. However, the protocol stipulated to include in the analysis all patients with EVAR-related adverse events, regardless of the duration of their follow-up. Patients with recorded mortality during follow-up, which was unrelated to the index or secondary EVAR procedure(s), were counted as lost to follow-up. Further clinical exclusion criteria are the presence of malignancy; autoimmune, inflammatory, or other serious systemic disease; and the presence of pre-existing or new-onset chronic renal failure (with eGFR < 45 mL/min/1.73m^2^), which limited the ability for long-term CTA follow-up. Finally, patients treated with an abdominal endograft with fewer than 10 total cases as well as patients lost to follow-up, for unknown reason, were also excluded.

### 2.1. Definition of Clinical Endpoints

Primary endpoint was considered the occurrence of any related complication (ARC), which included the following adverse events that could be related to the index or to reintervention EVAR procedure(s): Types Ia, Ib, III, and IV endoleaks, Type II endoleaks with AAA's sac growing, Migration Ia (proximal landing zone migration (PLZM)), Ib (distal landing zone migration (DLZM)), or both types, and stent graft's limb occlusions as well all EVAR-related reinterventions and mortality events. Secondary endpoints were considered all migration cases.

### 2.2. Definitions of Postimaging Data

The pre- and post-EVAR CTA imaging protocol was performed without per os Gastrografin uptake and with slice thickness 0.5 mm and interval 0.3 mm. Postimaging processing was based on analysis of Picture Archiving and Communication System (PACS) data with a dedicated 3D CTA analysis Horos software (Horosproject.org, sponsored by Nimble Co LLC d/b/a Purview, Annapolis, Maryland, United States). The standardized measurement technique of the studied iliac anatomo-morphological characteristics was based on analysis of anonymized PACS data using the center lumen line (CLL) technique and the 3D curved–MPR (multiplanar reformation) tool, with manual reconstruction of the CLL [14]. Each individual measurement in all CTAs was performed twice and blindly by two independent investigators (A.G.P. and D.A.C.). After intra- and interobserver variability analysis, the final value of each anatomical variable studied was calculated as the average of the four values resulting from the two blind measurements of the two observers. Our protocol stipulated that all potential differences > 10% between the two observers in any of the variables should be settled by a third senior investigator (G.A.P.) and his blinded measurement would serve as the final mean value.

The routine CTA analysis included all parameters necessary for daily clinical practice and especially the identification of any type of endoleak and migrations. Particularly in migrations, we identified two types: PLZM which was defined as the caudal displacement of the endograft ≥ 5 mm from its original position at the first-month CTA and DLZM defined as cephalad retraction of the endograft limb(s) ≥ 10 mm from its (their) original position on the first-month CTA [15]. All migrations with concomitant presence of Type Ia, Ib, III, and IV endoleaks were considered indications for reintervention. However, a PLZM alone was considered clinically important and indication for reintervention if the caudal displacement of the endograft exceeded ≥ 10 mm.

**Figure 1:**
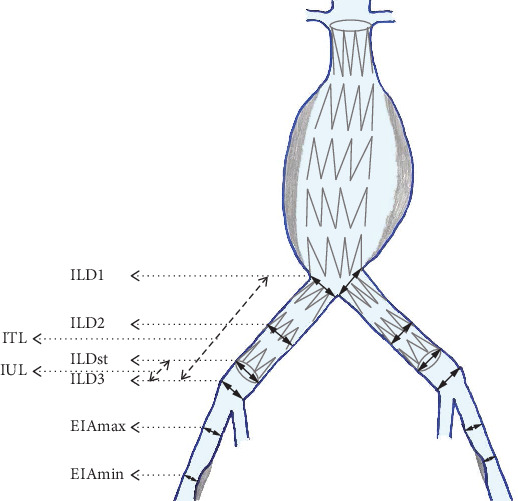
Protocol of measurements. Abbreviations: ILD = common iliac artery diameter, ITL = common iliac artery total length, IUL = length of uncovered from the stent graft part of the common iliac artery, EIAmax = external iliac artery maximum diameter, EIAmin = external iliac artery diameter at its narrowest point. In text and tables, the relevant abbreviations appear by adding to the beginning of the abbreviation the capital letter R or L for the right and left sides, respectively.

In addition to the routine CTA analysis, we further analyzed a total of eight primary variables, which were measured in postoperative CTAs, for all iliac arteries. The diameters of the iliac arteries were measured outer to outer wall (or adventitia to adventitia) [16] in the following six levels: (1) CIA at the aortoiliac bifurcation abbreviated as RILD1 for the right and LILD1 for the left side, (2) CIA at the middle of its length (abbreviated proportionally as RILD2 and LILD2), (3) CIA at iliac bifurcation (abbreviated proportionally as RILD3 and LILD3), (4) CIA at the end of stent graft's iliac limb (abbreviated as RILDst and LILDst), (5) external iliac artery (EIA) at the point of its greater diameter (abbreviated proportionally as REIAmax and LEIAmax), and (6) EIA at the point of its narrowest diameter (abbreviated proportionally as REIAmin and LEIAmin). We also measured the total length of the CIA defined as the length between the two bifurcations, from the aortoiliac to the CIA, which was abbreviated as RITL for the right side and LITL for the left, as well as the length of the uncovered iliac length between the end of implanted stent graft's iliac limbs and the CIA bifurcations, which were proportionally abbreviated as RIUL and LIUL. In pre-EVAR CTA data collection were obviously omitted the RILDst and LILDst diameter measurements as well as the RIUL and LIUL.  Figure 1 illustrates the topography of applied iliac artery CTA measurements.

In addition, to investigate the role of potential factors that may have influenced the evolution of the dimensions of the iliac arteries as well as to explore the effect of the alterations in the iliac arteries on the clinical outcomes, we calculated the following secondary variables: first is the absolute and percentage difference of the eight primary variables of the common iliac arteries, between the three studied postoperative time intervals, and second is the mean difference recorded in the eight primary variables of both common iliac arteries, between the same time intervals.

### 2.3. Statistical Analysis

All data were entered in a digital database and the statistical analysis was performed using the IBM SPSS Statistics software—version 28.0 for iOS (IBM Corp, Armonk, New York, United States). All statistical tests applied were two sided, and the level of statistical significance was set at *p* value < 0.05. The inter- and intraobserver variability was assessed by the Bland–Altman plot and regression analysis [17]. The agreement between the blind measurements of the same observer as well as between the two observers was proved excellent, and the option of the third senior observer was not used in any of the included patients. Categorical variables are presented as counts and percentages and were analyzed using the chi-squared test. Distribution of continuous variables was explored with normality plots and the Shapiro–Wilk test, and all were found to follow skewed distribution. Continuous variables of demographics are presented as median and interquartile range. Continuous variables from CTA analysis were analyzed by the nonparametric Wilcoxon signed rank test, as appropriate, and in tables and text are presented as mean ± stdv. Comparison of continuous CTA data between subgroups was based on two independent-sample Student's *t*-test and is presented as mean ± stdv. Independent categorical variables with a positive univariate association with the 5-year clinical outcomes were further analyzed with multivariate logistic regression analysis. Finally, the Kaplan–Meier analysis was used to construct survival plots and to assess the clinical outcomes during the follow-up.

## 3. Results

**Figure 2:**
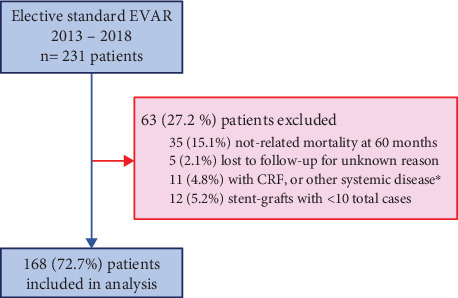
Flowchart of patients' enrollment in the study. Abbreviations: EVAR = endovascular aortic aneurysm repair, CRF = chronic renal failure. ⁣^∗^Other systemic disease included malignancy, autoimmune, inflammatory, or other serious systemic disease.

Figure 2 illustrates the study's enrollment flowchart. Sixty-three (27.2%) patients were excluded from the study, and 168 patients were finally enrolled for analysis. The demographics, baseline characteristics at the time of EVAR, and 30-day operative data are presented in Table 1.

### 3.1. Clinical Outcomes

The primary technical success was 100%. At the completion angiograms, we suspected in three patients (1.8%) a type of Ia endoleak, which was managed successfully, during the index procedure, with implantation of a proximal aortic cuff. No 30-day mortality or major morbidity was noted. A 30-day CTA follow-up revealed the presence of Type II endoleak in 16 (9.5%) patients and the absence of other types of endoleak and/or migration and confirmed the patency of bifurcated stent grafts.

During the first year, one (0.6%) patient was identified with proximal Type Ia endoleak and underwent a successful proximal cuff placement and there occurred three cases of stent graft's limb occlusion. In two limb occlusions, we performed a successful secondary intervention, while in one patient with mild gluteal claudication we followed conservative treatment, which led to slight clinical improvement over time. Additionally, five (31.3%) cases from the total of 16 Type II endoleaks detected at 30-day CTA were resolved completely during the first year.

At 24 months, three more Type II endoleaks were found to be spontaneously sealed but four new Type II cases were identified and two of these patients showed AAA's sac growth and required reintervention. Regarding the other types of endoleak, migrations, or any EVAR-related complications, no further events were recorded during the 24 months of follow-up. Conversely, in the period between the second and fifth year, 14 (8.3%) new EVAR-related adverse events occurred. In all cases, a significant migration of the stent graft was noted. In seven (4.2%) patients, a clinically important PLZM was detected, which was associated with an increase in diameter of the proximal aortic neck. One (0.6%) patient showed DLZM alone, with retraction of both stent graft iliac limbs, while in six (3.6%) patients the DLZM was combined with clinically important PLZM. Nine (5.4%) of these patients had also a Type Ia endoleak, three (1.8%) of them had Type Ib endoleak, and in one (0.6%) patient, a Type III endoleak was detected. Finally, one (0.6%) patient with clinically important PLZM had a concomitant Type II endoleak that was firstly detected as a new-onset endoleak at 24 months and remained visible until the fifth year, with no increase in sac diameter. At 60 months of follow-up, three additional Type II endoleaks were detected with stable AAA's sac maximum diameter. Apart from the two patients with sac growth at 24 months, all Type II endoleaks were managed conservatively, and finally, 13 patients (7.7%) had a persistent Type II endoleak, which was still present even after 5 years, although none of them was associated with increase in AAA's sac diameter.

**Figure 3:**
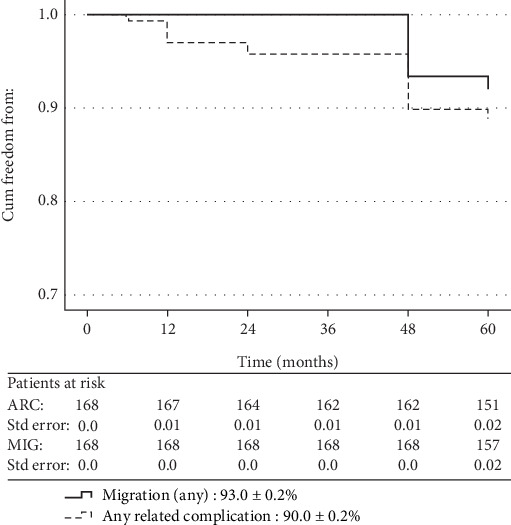
Kaplan–Meier curves at 60 months of any type of migration and of any related complication.

The total, related to index or reintervention procedures, mortality, of the entire series, was 2.4% (*n* = 4), and all events occurred at/or after the 60-month CTA follow-up.  Table 2 summarizes the clinical outcomes during the follow-up, and Figure 3 shows the Kaplan–Meier survival analysis of primary and secondary endpoints. The (descriptive) incidence of ARC primary endpoint at 2 years of follow-up was 3.6% (*n* = 6) and at 5 years reached the total of 20 cases (11.9%). No migration cases were identified at 2 years, but 14 (8.3%) migrations were detected at 5 years.  Table 3 shows the univariate analysis of 5-year clinical outcomes in relation with the type and characteristics of implanted stent grafts. The suprarenal proximal fixation (*n* = 146) was significantly associated with better outcomes in terms of ARC and migrations (*p* = 0.017 and *p* = 0.009, respectively) compared with those with infrarenal proximal fixation (*n* = 22). Similarly, better results (relevant *p* values 0.007 and 0.006, respectively) were recorded for the subgroup of suprarenal fixation with hooks (*n* = 105) compared with the subgroup of infrarenal or suprarenal without hook fixation (*n* = 63). However, although the multivariate logistic regression (Table 4) confirmed a trend in favor of subgroup with suprarenal fixation with hooks, it failed to show a definite association of these subgrouping variables with worse clinical outcomes.

### 3.2. Common Iliac Artery Remodeling

The mean iliac diameters and length increased at the first month by 0.11 and 0.08 mm, respectively (Table 5, *p* < 0.001 in all variables).

**Figure 4:**
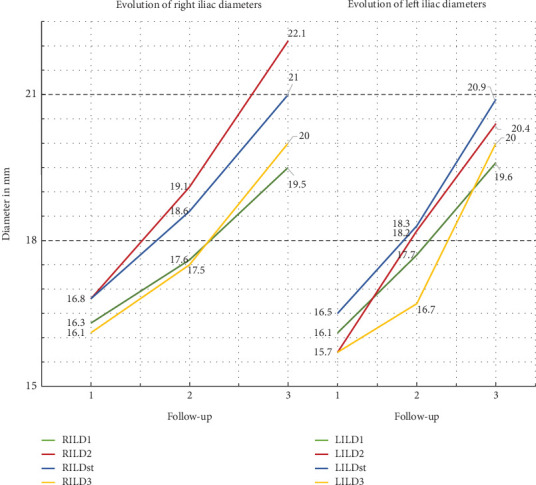
Graph of the evolution of the diameters' measurements of the common iliac artery over time. Abbreviations: ILD = common iliac artery diameter. Adding the capital letter R or L to the beginning of the abbreviation indicates the right and left sides, respectively.

All CIA diameters increased significantly (*p* < 0.001) and progressively in all time periods of the study.  Table 6 summarizes the analysis of mean ± stdv values of CTA measurements and the comparison between them. At 24 months, the highest rate of increase in iliac diameter was seen in LILD2, which showed an increase of 15.9%, while at the same time in LILD4 was recorded the lower increase percent rate of 6.4%. The mean rate of progression of all eight common iliac diameters at 2 years was 9.7%. However, the relevant mean percent increase rate at 60 months in iliac diameters was 22.8% (minimum 19.5% in RILD1 and maximum 31.8% in RILD2). The average difference (increase) rate of the period from 24 to 60 months was 11.7%. At this time interval, the lowest evolution rate with an increase of 11.0% was observed in RILD1, while the highest rate of 19.2% progression was observed in LILD4 which had shown the lowest rate of increase (6.4%) in the first 2 years.  Figure 4 shows the graph of the evolution of the common iliac artery diameters during the 60 months of follow-up. The mean, monthly, absolute increase for the first 24 postoperative months was 0.07 mm and remained the same for the next 36 months, at the end of follow-up. Univariate analysis of factors potentially associated with the evolution in common iliac artery dimensions (Table 7) revealed that the extreme iliac oversizing (≥ 20%) was associated with greater percent rate of difference in all right iliac diameters between the first- and 60th-month CTA measurements. However, the relative comparison for the left side measurements failed to show a similar positive association of extreme oversizing with the long-term alterations in common iliac diameters. Similarly (Table 7), no association was found with the percent rate of differences in all iliac diameters if the subgrouping variable was the length of uncovered segment (> 15 mm or ≤ 15 mm) of iliac artery in index EVAR procedure.

**Figure 5:**
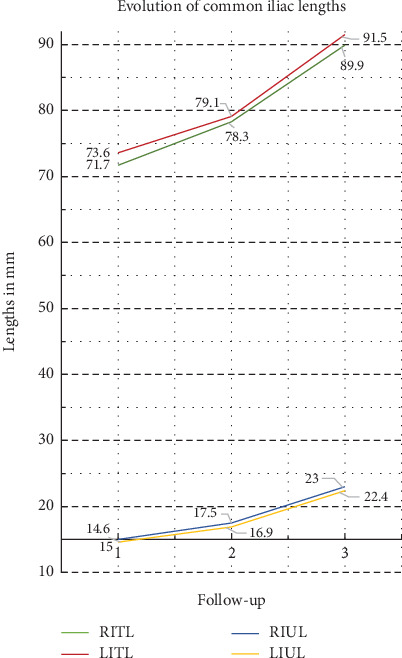
Graph of the evolution of the lengths' measurements of the common iliac artery over time. Abbreviations: ITL = common iliac artery total length, IUL = length of uncovered from the stent graft part of the common iliac artery. Adding the capital letter R or L to the beginning of the abbreviation indicates the right and left sides, respectively.

Significant increase was also recorded in all common iliac length measurements at all time intervals (*p* < 0.001, Table 6). At 60 months of follow-up, the length of the right common iliac artery increased by 18.2 mm (from 71.7 ± 15.5 to 89.9 ± 19.0 mm, 25.4% increase, *p* < 0.001) and the left by 17.9 mm (from 73.6 ± 16.1 to 91.5 ± 19.7 mm, 24.4% increase, *p* < 0.001). At the same time, the uncovered from stent graft segments of the right and left common iliac arteries increased by 53.6% (absolute difference 8.0 m) and 54.1% (absolute difference 7.8 mm), respectively, *p* value < 0.001 in both variables. The respective mean rates of increase at 24 months of total and uncovered iliac artery lengths were similar (15.3% and 32.0%, respectively), and the graph of Figure 5 highlights that the length variables showed an almost linear progression over time. The recorded mean absolute increase in common iliac length was 0.26 mm/month at the period first to 24th month and reached the value of 0.34 mm/month at the time interval from 24th to 60th month. The respective values for the evolution of common iliac artery uncovered segment were 0.10 and 0.15 mm/month. Subgrouping analysis (Table 7) of extreme oversizing and > 15 mm uncovered iliac length showed a significant difference in percent rate of length increase only in the left CIA's uncovered segment.

#### 3.2.1. Impact of Common Iliac Artery Remodeling in Outcomes

The mean difference between the first- and 60th-month CTA measurements of both iliac arteries and the type of proximal aortic fixation, which were suggested from univariate analysis to have a potentially positive association with worse clinical outcomes, were entered in a multivariate regression analysis model (Table 8), which revealed that increasing difference in the diameter of distal iliac landing zone (ILD3) was 2.8 times more likely to exhibit ARC, but suprarenal proximal aortic fixation with hooks was associated with a significant reduction in probability of ARC's occurrence.

### 3.3. Evaluation of External Iliac Diameters

Conversely with the common iliac diameters, the comparison of external iliac artery diameters (Table 6) showed a different pattern of changes. The maximum diameter values showed a small mean increase in maximum diameter by 6.1% (absolute mean increase 0.65 mm) at 5 years, while in relevant minimum diameters was recorded a 2.6% decrease over time (absolute mean decrease 0.3 mm).

## 4. Discussion

The long-term outcome of an EVAR procedure is closely related to the anatomic characteristics of the AAA and the selection of the appropriate optimal stent graft for the particular patient's AAA morphology [18]. A growing literature suggests that proper oversizing and adequate as well as time-resistant sealing in the proximal and distal sealing zones are essential to minimize the post-EVAR serious complications associated with stent grafting and especially migrations and endoleaks [6, 8, 19]. Moreover, it is true that the primary role in long-term complications belongs to the proximal aortic sealing zone, and this makes obvious the reason why this topic has almost monopolized the relative interest of the vascular surgery research community [9–12, 20, 21]. However, DLZF is a factual problem of clinical practice; the consequences of which can be as serious as PLZF, which can equally compromise the long-term success of EVAR, and there is a gap in the current published knowledge [22, 23]. The published experience consists mainly of small retrospective series, reporting outcomes using outdated stent grafts, with incidental reporting on the iliac remodeling data within a larger EVAR reference frame, and/or with short- to medium-term follow-up [24–30].

The current study represents the first research attempt in the literature, focused exclusively on iliac artery remodeling occurring in the long-term post-EVAR period, using contemporary bifurcated aortic grafts and with prospective collection of data. In order to better understand the alterations in the dimensions of the iliac arteries, we established a research protocol with measurements of the iliac arteries' diameters at four levels. We also, for the first time, introduced the routine measurement of two iliac length parameters that we believe best represent the long-term geometric remodeling of the iliac vessels.

Our data support that there is an ongoing, progressive, significant, and almost linear evolution, in the meaning of increase, in all common iliac diameters and lengths. We recorded, at all-time intervals, a common pattern of remodeling in both iliac arteries. The greater diameter increase, approximately 31% at 5 years, was observed in the middle of iliac arteries, which level usually corresponds with the greater preoperative diameter of iliac arteries if they had been affected with aneurysmatic disease. The mean absolute rate of increase in diameters remained impressively constant over time and was calculated to be 0.07 mm per month, while the mean percentage increase, between all four levels of measurements and both iliac arteries, was 11.7% at 24 months and 22.8% at 60 months. Considering that the recommended oversizing of iliac arteries is at the range between the ideal > 10% and excessive < 15%, the 22.8% of diameter increase at 60-month follow-up might represent a source of potential DLZF at a later time, beyond the fifth year. Subgroup analysis in our series failed to show a clear association of even extreme oversizing > 20% with post-EVAR dilatation of iliac arteries. Proportionally significant, progressive, and linear were the alterations observed in the measured CIA lengths. The elongation of the total length of the common iliac artery reached the considerable rate of 0.26 m per month for the first 24 months and exceeded the value of 0.34 mm per month for the next 36 months (mean percent rates 8.4% and 15.3%, respectively). The recorded absolute elongation of common iliac artery's uncovered segment at the same time intervals was 0.10 and 0.15 mm per month, but the overall percentage of increase at 60 months was up to 53.9%, which was not affected from the extreme oversizing (> 20%) or from the increased (> 15 mm) distance of the end of implanted iliac limbs from the iliac bifurcation at index procedure. Our findings, in terms of both iliac dimension parameters, diameters and lengths, suggest that there is an ongoing aneurysmal process that is not directly related to the characteristics of the implanted stent grafts. This process may be slow in pace but continues to elongate and dilate the iliac arteries over time, exposing them to a continuous hemodynamic stress with unknown future consequences, particularly in younger patients with longer life expectancies.

Our multivariate analysis of factors potentially affecting the clinical outcomes revealed that the single independent factor, related with the technique of index EVAR procedure, which was associated with fewer EVAR-related complications was the suprarenal proximal aortic fixation with hooks. Entering this variable into the multivariate regression model along with the mean difference of all measured dimension parameters revealed that dilatation of the iliac artery at the distal landing zone increases considerably, by 2.8 times, the likelihood of facing an EVAR-related complication. These findings do not support that infrarenal fixation may be generally the reason for an EVAR failure, because our series did not include any patients treated with the Gore Excluder (W. L. Gore & Associates, Newark, Delaware, United States) stent graft, which has an impressive record of reported successful long-term standard EVARs [31]. However, the three brands of AAA stent grafts included in our series, with the common characteristic of suprarenal fixation with hooks, shared a similar design platform and fabric lining with nitinol exoskeleton, and undoubtfully, in our experience, their use was associated with better long-term outcomes [32–34].

Our study shared the same limitations with the single-center observational studies [35]. The number of included patients, although considerable, is relatively small to draw firm conclusions and the series represent the experience of a single vascular surgeon. The data were collected prospectively but our analysis is retrospective, and this could raise the question of potential selection bias. On the other hand, we tried to be as fair as it was possible keeping the overall CTA measurement process completely blind and methodologically objective. Furthermore, our series consisted of consecutive patients, which were treated and followed with, as it was possible, a common protocol and with an increased homogeneity of our data.

## 5. Conclusions

The degenerative aneurysmal process continues to affect the wall of the common iliac arteries over time, resulting in significant dilatation and elongation of all segments of the iliac arteries, which was associated with worsening EVAR outcomes. This ongoing affection of iliac arteries should be considered when sizing and planning a standard EVAR, as it appears to be associated with long-term failures, and this underscores the importance and necessity of unimpaired follow-up even after 5 years from the index procedure, in order to maintain the long-term safety and efficacy of EVAR.

## Figures and Tables

**Table 1:** Baseline demographics, preoperative clinical status, and 30-day operative data, presented as *n* (%) or median (interquartile range).

	**Total ** **n** = 168
Demographics	
Age (years)	72.0–9.0
Male gender	162–96.4
Preoperative clinical status	
Hypertension	152–90.5
Diabetes	63–37.5
Dyslipidemia	147–87.5
Coronary artery disease	70–41.7
Chronic respiratory disease	19–11.3
Smoking	114–67.9
Peripheral arterial disease	16–9.5
Body mass index > 30	25–14.9
Antiplatelet	103–61.3
Statin	106–63.1
30-day operative data	
EVAR operative time (min)	68.0–25.0
EVAR fluoroscopy time (min)	11.0–6.0
Contrast medium (mL)	178.0–60.0
Locoregional anesthesia	159–94.6
30-day mortality/morbidity	0.0–0.0
30-day endoleak Types I, III, or IV	0.0–0.0
30-day endoleak Type II	16–9.5
Primary technical success	168–100.0

**Table 2:** Clinical follow-up data.

**n** = 168	**1st year (** **n** **, %)**	**2nd year (** **n** **, %)**	**5th year (** **n** **, %)**
Endoleak Type Ia	1–0.6	0	9–5.4
Endoleak Type Ib	0	0	3–1.8
Endoleak Types III or IV	0	0	1–0.6
Endoleak Type II^a^	0	2^b^–1.2	0
Total endoleak Types Ia, Ib, III, IV, and II^a^	1–0.6	2–1.2	13–7.7
Migration at the proximal aortic neck	0	0	7–4.2
Migration/retraction of iliac limbs	0	0	1–0.6
Both types of migration	0	0	6–3.6
Total migrations	0	0	14^d^–8.3
Rupture	0	0	3–1.8
Limb occlusion	3^c^–1.8	0	0
Related reintervention	3–1.8	2–1.2	14^d^–8.3
Related mortality	0	0	4–2.4
All related complication events	4^c^–2.4	2–1.2	14–8.3
Endoleak Type II^e^	11–6.5	12^b^–7.1	13–7.7

^a^Type II endoleak with AAA's sac growth.

^b^Four new-onset Type II endoleaks were detected at 24 months, and two of them required reintervention.

^c^Two patients with limb occlusion had also Type II endoleak; in one patient, the limb occlusion was treated conservatively.

^d^All migrations (two with rupture) underwent reintervention, 13 of them also showed Types Ia, Ib, or III/IV endoleaks, and one patient also had a Type II endoleak detected previously at 24 months.

^e^Total Type II endoleaks pictorially evident at each time frame.

**(a):** 

**Endografts**	**n** **, %**	**ARC** ^a^	**p**	**Migration** ^b^	**p**
		**(20–11.9)**		**(14–8.3)**	
Endurant	58–34.5	3–5.2	0.65	2–3.4	**0.024**
E-tegra	28–16.7	2–7.1		1–3.6	
Treo	19–11.3	2–10.5		1–5.3	
Anaconda	22–13.1	6–27.3		5–17.2	
AFX2	29–17.3	6–20.7		5–17.2	
Ovation	12–7.1	1–8.3		0–0	
Total	168–100	20–11.9		14–11.9	

**(b):** 

**Endograft features**	**n** **, %**	**p**
	Any related complication (20–11.9)	
Liner material		
ePTFE (*n* = 41)	7–17.1	0.240
Dacron (*n* = 127)	13–10.2	
Proximal fixation level		
Suprarenal (*n* = 146)	14–9.6	**0.017**
Infrarenal (*n* = 22)	6–27.3	
Type of proximal fixation		
Suprarenal with hooks (*n* = 105)	7–6.7	**0.007**
Infrarenal or suprarenal without hooks (*n* = 63)	13–20.6	
	Migration Ia, Ib, or both types^b^ (14–8.3)	
Liner material		
ePTFE (*n* = 41)	5–12.2	0.303
Dacron (*n* = 127)	9–7.1	
Proximal fixation level		
Suprarenal (*n* = 146)	9–6.2	**0.009**
Infrarenal (*n* = 22)	5–22.7	
Type of proximal fixation		
Suprarenal with hooks (*n* = 105)	4–3.8	**0.006**
Infrarenal or suprarenal without hooks (*n* = 63)	10–15.9	

*Note:* The boldface data indicates statistical significance at *p* levels < 0.05.

^a^ARC = any related with index EVAR or secondary intervention complication, including Types Ia, Ib, III, and IV endoleaks; Type II endoleaks requiring reintervention; Migration Type Ia, Ib, or both types; all secondary procedures related to index EVAR procedures; and all mortality events that were associated to the index EVAR or related secondary procedures.

^b^Cases with Type Ia, Ib, III, and IV endoleak were almost identical with migration, with the exception of one case of Ia migration in a patient with Type II endoleak, who was diagnosed at 12 months of follow-up and underwent secondary procedure due to increase of AAA's sac diameter in the 48th month.

**Table 4:** Multivariate logistic regression analysis of potential (from univariate analysis) predictors of any related complication and migration.

	**Sig. (** **p** **)**	**Exp (** **B** **)**	**95% CI for Exp (** **B** **)**
			**Lower–upper**
	Any related complication (*n* = 20–11.9)		
Proximal fixation level			
Suprarenal (*n* = 146)	0.344	0.549	
vs.			0.159–1.901
Infrarenal (*n* = 22)			
Type of proximal fixation			
Suprarenal with hooks (*n* = 105)	0.063	0.347	
vs.			0.113–1.061
Infrarenal or suprarenal without hooks (*n* = 63)			
	Migration Ia, Ib, or both types (*n* = 14–8.3)		
Proximal fixation level			
Suprarenal (*n* = 146)	0.282	0.472	
vs.			0.120–1.853
Infrarenal (*n* = 22)			
Type of proximal fixation			
Suprarenal with hooks (*n* = 105)	0.072	0.285	
vs.			0.073–1.121
Infrarenal or suprarenal without hooks (*n* = 63)			

**Table 5:** Mean ± stdv values of CTA measurements of iliac diameters and lengths and two related sample analysis between preoperative and first-month CTAs.

	**Pre-op**	**1st month**	**p**	**Absolute difference**	**Mean evolution**
Common iliac diameters					
RILD1	16.2 ± 2.7	16.3 ± 2.7	< 0.001	0.12	0.11
RILD2	16.6 ± 3.7	16.8 ± 3.7	< 0.001	0.08	
RILD3	16.0 ± 3.3	16.1 ± 3.3	< 0.001	0.11	
LILD1	16.0 ± 2.7	16.1 ± 2.7	< 0.001	0.11	
LILD2	15.5 ± 3.3	15.7 ± 3.3	< 0.001	0.12	
LILD3	15.6 ± 2.9	15.7 ± 2.9	< 0.001	0.12	

Common iliac lengths					
RITL	71.6 ± 15.5	71.7 ± 15.5	< 0.001	0.07	0.08
LITL	73.5 ± 16.1	73.6 ± 16.1	< 0.001	0.08	

*Note:p* value calculated with the Wilcoxon signed rank test.

Abbreviation: Pre-op; preoperative.

**Table 6:** Mean ± stdv values of postoperative CTA measurements of iliac diameters and lengths and *p* values of two related sample analysis by the Wilcoxon signed rank test.

	**1st month**	**24th month**	**60th month**	**1 vs. 24** **p**–%**diff**	**24 vs. 60** **p**–%**diff**	**1 vs. 60** **p**–%**diff**
*Common iliac diameters*						
RILD1	16.3 ± 2.7	17.6 ± 2.8	19.5 ± 3.4	< 0.001–7.6	< 0.001–11.0	< 0.001–19.5
RILD2	16.8 ± 3.7	19.1 ± 4.3	22.1 ± 4.8	< 0.001–13.8	< 0.001–15.8	< 0.001–31.8
RILDst	16.8 ± 3.8	18.6 ± 4.3	21.0 ± 4.7	< 0.001–11.2	< 0.001–13.0	< 0.001–25.7
RILD3	16.1 ± 3.3	17.5 ± 32.8	20.0 ± 4.2	< 0.001–8.5	< 0.001–15.9	< 0.001–25.7
LILD1	16.1 ± 2.7	17.7 ± 3.1	19.6 ± 3.6	< 0.001–9.9	< 0.001–11.2	< 0.001–22.2
LILD2	15.7 ± 3.3	18.2 ± 4.2	20.4 ± 4.5	< 0.001–15.9	< 0.001–12.0	< 0.001–30.2
LILDst	16.5 ± 3.4	18.3 ± 4.1	20.9 ± 4.5	< 0.001–10.9	< 0.001–14.1	< 0.001–27.1
LILD3	15.7 ± 2.9	16.7 ± 3.1	20.0 ± 3.9	< 0.001–6.4	< 0.001–19.2	< 0.001–27.6
***Mean percent evolution rate***				***9.7***	***11.7***	***22.8***
*Common iliac lengths*						
RITL	71.7 ± 15.5	78.3 ± 16.5	89.9 ± 19.0	< 0.001–9.3	< 0.001–14.7	< 0.001–25.4
LITL	73.6 ± 16.1	79.1 ± 17.1	91.5 ± 19.7	< 0.001–7.5	< 0.001–15.8	< 0.001–24.4
***Mean percent evolution rate***				**8.4**	**15.3**	**24.9**
RIUL	15.0 ± 6.7	17.5 ± 8.2	23.0 ± 10.9	< 0.001–16.5	< 0.001–31.8	< 0.001–53.6
LIUL	14.6 ± 7.8	16.9 ± 9.4	22.4 ± 12.7	< 0.001–16.5	< 0.001–32.2	< 0.001–54.1
***Mean percent evolution rate***				***16.5***	***32.0***	***53.9***
*External iliac diameters*						
REIAmax	10.8 ± 2.0	10.9 ± 2.1	11.4 ± 2.1	0.165–1.0	< 0.001–4.5	< 0.001–5.5
LEIAmax	10.9 ± 2.3	10.8 ± 2.1	11.6 ± 2.4	0.083 to (−0.7)	< 0.001–7.3	< 0.001–6.6
REIAmin	9.4 ± 2.0	9.4 + 2.4	9.1 ± 1.9	0.001–0.4	< 0.001 to (−2.5)	0.003 to (−2.1)
LEIAmin	9.3 ± 2.0	8.9 ± 2.0	9.0 ± 2.0	< 0.001 to (−4.1)	0.302–1.2	< 0.001 to (−3.0)

*Note:p*–%diff: *p* = *p* value of the Wilcoxon signed rank test and %diff = percent difference between the values; negative percentage difference observed at certain intervals at diameters of the external iliac appears in parentheses. The boldface data indicates statistical significance at *p* levels < 0.05.

**Table 7:** The impact of oversizing and length of the uncovered iliac artery in the evolution of common iliac artery dimensions over time. Assessment of evolution was based on percent differences between measurements in CTAs at the first and 60th month^ab^.

	**Oversizing**			**Length of uncovered iliac artery**		
	**≥ 20%**	**< 20%**		**> 15** mm	**≤ 15 mm**	
*Right common iliac*						
	**n** = 63	**n** = 105	**p**	**n** = 95	**n** = 73	**p**
RILD1	22.6% ± 13.9%	18.4% ± 11.6%	0.020	21.1% ± 11.8%	18.5% ± 13.5%	0.095
RILD2	33.7% ± 10.7%	31.1% ± 8.3%	0.037	32.7% ± 9.8%	31.2% ± 8.6%	0.157
RILDst	28.1% ± 9.6%	24.8% ± 8.6%	0.010	26.9% ± 8.5%	24.9% ± 9.8%	0.078
RILD3	27.9% + 9.3%	24.6% ± 8.4%	0.010	27.1% ± 8.3%	25.1% ± 7.4%	0.082
RITL	25.9% ± 7.8%	25.7% ± 7.6%	0.419	25.3% ± 7.4%	26.5% ± 7.9%	0.160
RIUL	52.3% ± 12.9%	53.6% ± 13.1%	0.274	53.7% ± 12.3%	53.2% ± 13.8%	0.250
*Left common iliac*						
	**n** = 92	**n** = 76	**p**	**n** = 68	**n** = 100	**p**
LILD1	22.8% ± 12.9%	22.1% ± 12.3%	0.377	23.3% ± 12.5%	21.9% ± 12.7%	0.244
LILD2	30.5% ± 10.1%	30.3% ± 8.4%	0.422	29.9% ± 7.6%	30.8% ± 10.4%	0.273
LILDst	27.5% ± 9.2%	27.0% ± 9.0%	0.378	27.5% ± 8.6%	27.2% ± 9.5%	0.422
LILD3	27.9% ± 9.2%	27.5% ± 9.0%	0.377	27.9% ± 8.6%	27.6% ± 9.5%	0.421
LITL	25.0% ± 8.3%	24.5% ± 6.7%	0.313	24.2% ± 7.0%	25.2% ± 7.9%	0.200
LIUL	49.6% ± 11.5%	56.6% ± 14.0%	< 0.001	55.2% ± 13.1%	51.1% ± 13.0%	0.023

^a^Similar were the results if the compared variables were the absolute measurements' differences at the same time intervals (data not shown).

^b^Similar were the results if the subgrouping variables were the coexistence of both technical features of oversizing ≥ 20% and length of uncovered iliac artery > 15 mm, which was observed in 38 patients in the right side, in 28 patients in the left side, and in 6 patients in both sides (data not shown). Analysis by two independent-sample Student's *t*-test.

**Table 8:** Multivariate regression analysis of effects in clinical outcomes using as factors the mean differences (increase) in both iliac artery dimensions between the first- and 60th-month CTAs and the type of proximal aortic fixation.

	**Any related complication**			
	**M** **e** **a** **n** ± **stdv**	**p**	**Exp (** **B** **)**	**95% CI**
Mean difference of both iliac arteries				
ILD1	3.37 ± 2.0	**0.023**	0.412	0.192–0.885
ILD2	5.03 ± 1.7	**0.035**	0.370	0.147–0.934
ILDst	4.38 ± 1.6	0.129	2.786	0.742–10.460
ILD3	4.24 ± 1.5	0.287	1.746	0.625–4.879
ITL	18.10 ± 6.0	0.273	0.949	0.863–1.043
IUL	7.95 ± 4.5	0.275	1.055	0.958–1.162
Type of proximal fixation	**n** ** , %**	**0.021**	0.288	0.100–0.831
Suprarenal with hooks	105–62.5			
Infrarenal or suprarenal without hooks	63–37.5			

*Note:* The statistical model correctly classified 89.3% of cases. Increasing difference in the diameter of the distal iliac landing zone (ILD3) was 2.78 times more likely to exhibit (any) EVAR-related complication. Increasing differences in ILD1 and ILD12 were independent predictors of worse clinical outcomes, but suprarenal proximal aortic fixation with hooks was associated with a significant reduction in the likelihood of exhibiting (any) EVAR-related complication. ILD(1,2,st,3) = mean iliac artery diameter at the points of measurements of primary variables. The values were calculated as the mean between both common iliac arteries. The boldface data indicates statistical significance at *p* levels < 0.05.

## Data Availability

All data will be available upon request to any researcher who wishes to access our data and for any reason including verification of our results.
